# MAUDE Registry Analysis of Pericardial Effusions Following Watchman and Amulet Left Atrial Appendage Occlusion

**DOI:** 10.1016/j.jacadv.2026.102789

**Published:** 2026-05-15

**Authors:** Sneha S. Limaye, Karl Q. Schwarz, Mehmet K. Aktas, Frederick S. Ling

**Affiliations:** a University of Rochester Medical Center, Rochester, New York, USA; b Louis Stokes Veterans Affairs Medical Center, Cleveland, Ohio, USA

**Keywords:** amulet, LAAO, pericardial effusion, watchman

## Abstract

**Background:**

Percutaneous left atrial appendage occlusion (LAAO) devices are an important therapeutic alternative to oral anticoagulation for stroke prevention in patients with nonvalvular atrial fibrillation. In patients undergoing LAAO, a pericardial effusion is the most common serious complication, with some effusions occurring in a delayed manner.

**Objectives:**

The purpose of this study was to investigate the incidence and outcomes of acute pericardial effusion vs late pericardial effusion (LPE) following LAAO device implant in those who develop pericardial effusion.

**Methods:**

The Manufacturer and User Facility Device Experience database consists of deidentified, public information. The terms “Amplatzer Amulet” were searched using the simple search method. A separate search for the term “Watchman” was performed using the same method. The results include data beginning at the time of the device’s Food and Drug Administration approval through December 2024. Reports were selected if a pericardial effusion occurred.

**Results:**

In the Manufacturer and User Facility Device Experience database, 290 pericardial effusions were reported among patients receiving an Amulet LAAO device compared to 33 reported cases for those receiving a Watchman device. Among Amulet LAAO implants who developed a pericardial effusion, 99/290 (34.1%) were LPEs; 2/33 (6.1%) were LPEs in Watchman LAAO implants who developed pericardial effusion. Of patients who had Amulet implant and developed LPE, 54/99 (54.5%) needed intervention; of those with acute pericardial effusion, 123/191 (64.4%) needed intervention.

**Conclusions:**

These observations suggest that LPEs adversely affect patient outcomes and can cause significant morbidity. These findings highlight the importance of long-term surveillance of patients for complications after successful LAAO.

Atrial fibrillation (AF) is the most common cardiac arrhythmia in adults and is associated with a higher stroke risk that is mitigated by the initiation of oral anticoagulants (OACs).^1^ However, in certain patients a variety of factors prohibit the safe initiation and maintenance of OAC, such as major bleeding, higher relative risk for major bleeding based on the HAS-BLED score, fall risks, and comorbidities. Percutaneous left atrial appendage occlusion (LAAO) has emerged as an important therapeutic alternative to OAC for stroke prevention in patients with nonvalvular AF. Recent guidelines from the American College of Cardiology and American Heart Association provide a level IIA recommendation for LAAO in patients with nonvalvular AF requiring anticoagulation but who do not tolerate anticoagulation.^2^

The Boston Scientific Watchman device was approved for LAAO on March 13, 2015, by the Food and Drug Administration (FDA) for stroke prevention in patients with nonvalvular AF.^3^ The Watchman device has since undergone several iterations, with the most current implanted model being the Watchman FLX Pro.^4^ The Abbott Amplatzer Amulet device was similarly approved by the FDA on August 16, 2021.^5^ There have been reports of late-presenting pericardial effusion (PE) (LPE) occurring weeks to months after the procedure in patients undergoing LAAO with an Amulet device.^6^ Several case reports describe erosion and perforation of the Amulet device into the pulmonary artery (PA).^7^ The incidence of LPE following LAAO has not been well described.

## Methods

The study population consisted of data logged in the Manufacturer and User Facility Device Experience (MAUDE) database. The MAUDE database includes reports of adverse events involving medical devices. The MAUDE database consists of deidentified, public information, and this study was deemed exempt from the University of Rochester Institutional Review Board review. The terms “Amplatzer Amulet” were searched using the simple search method. A separate search for the term “Watchman” was performed using the same method. The results include data beginning at the time of the device’s FDA approval through December 2024. Reports were selected if a PE was described.

### Data collection and measurements

Each report in the MAUDE database has a corresponding free-text event description. These descriptions are typically reported by the implanting physician and are of variable detail. The descriptions pertaining to PE were extracted and reviewed. An acute PE (APE) was defined as occurring in-hospital. LPE was defined as occurring following hospital discharge. The mortality endpoint consisted of all-cause deaths as reported in each event description.

### Statistics

Given the use of the MAUDE database, analyses were limited to descriptive statistics. Adverse event reports for each device were summarized using counts and percentages.

Inferential statistical analyses, including incidence rates, CIs, and comparative modeling, were not performed because MAUDE is a passive surveillance system that does not provide reliable denominator data on total device use. Therefore, findings are presented as frequencies of reported events rather than population-based event rates.

## Results

### Amulet implants

Among 290 patients with an Amulet device who developed a PE, 191/290 patients (65.9%) developed an APE, and 99/290 patients (34.1%) developed an LPE. A pericardiocentesis was performed in 103/191 (53.9%) patients with an APE and in 46/99 patients (46.5%) with an LPE. A pericardial window was performed in 3/191 (1.6%) patients with an APE and in 6/99 (6.1%) patients with an LPE (Table 1). An exploratory sternotomy or thoracotomy was required in 17/191 (8.9%) patients who experienced an APE and in 2/99 (2.0%) patients with an LPE. Overall, with intervention defined as pericardiocentesis, pericardial window, or exploratory sternotomy, 123/191 (64.4%) patients with APE needed intervention and 54/99 (54.5%) patients with LPE needed intervention. Death was reported in 16/191 (8.4%) patients with an APE, and in 8/99 (8.0%) patients with an LPE.

**Table 1:** Reported Outcomes and Intervention Characteristics of 290 Patients Who Received an Amulet Device Implant and Developed Pericardial Effusion

Outcomes and Intervention	Acute In-Hospital Pericardial Effusion (n = 191, 65.9%)	Late-Presenting Pericardial Effusion (n = 99, 34.1%)
Pericardiocentesis∗	103 (53.9%)	46 (46.5%)
Operating room	17 (8.9%)	2 (2.0%)
Pericardial window	3 (1.6%)	6 (6.1%)
Effusions requiring intervention	123 (64.4%)	54 (54.5%)
Alive	175 (91.6%)	91 (91.9%)
Deceased	16 (8.4%)	8 (8.0%)
Pulmonary artery involvement	10 (5.2%)	2 (2.0%)

∗1 patient with APE underwent pericardiocentesis, pericardial window, and other cardiac surgery.

∗1 patient with APE underwent pericardiocentesis and pericardial window.

∗1 patient with LPE underwent pericardiocentesis and pericardial window.

∗ 14 patients with APE underwent pericardiocentesis and cardiac surgery (nonpericardial window).

In the APE group, 10/191 patients (5.2%) were suspected to have developed a PE due to PA injury, with APE occurring immediately postprocedure; 2/99 (2.0%) in the LPE group were suspected to have PA injury. Among those with PA injury, 5 patients died, underscoring the high mortality rate associated with PA injury-related PEs.

### Watchman implants

Among the 33 patients with a Watchman device who developed a PE, 31/33 patients (93.9%) developed an APE and 2/33 (6.1%) developed an LPE (Table 2). Among those with an APE, 2/31 died and in those with an LPE, 1/2 died. A pericardiocentesis was performed in 22/31 (71.0%) patients with an APE and in 0 patients with an LPE. One patient with LPE required pericardial window. A total of 11/31 patients (35.5%) who experienced an APE underwent exploratory sternotomy or thoracotomy. Overall, an intervention consisting of pericardiocentesis, pericardial window, or exploratory thoracotomy and/or sternotomy, was reported in 24/31 (77.4%) of patients with APE and 1/2 patient with an LPE.

**Table 2:** Reported Outcomes and Intervention Characteristics of 33 Patients Who Received a Watchman Device Implant and Developed Pericardial Effusion

Outcomes and Intervention	Acute In-Hospital Pericardial Effusion (n = 31, 93.9%)	Late-Presenting Pericardial Effusion (n = 2, 6.1%)
Pericardiocentesis∗	22 (71.0%)	0 (0.0%)
Operating room	11 (35.5%)	0 (0.0%)
Pericardial window	0 (0.0%)	1 (50.0%)
Pericardiocentesis and surgery	9 (29.0%)	0 (0.0%)
Effusions requiring intervention	24 (77.4%)	1 (50.0%)
Alive	29 (93.5%)	1 (50.0%)
Deceased	2 (6.5%)	1 (50.0%)
Pulmonary artery involvement	0 (0.0%)	0 (0.0%)

∗ 9 patients with APE underwent pericardiocentesis and cardiac surgery (nonpericardial window).

The most common presentation reported by patients with an Amulet device who developed an LPE was chest pain (Table 3). Both patients who developed an LPE after Watchman implant presented with chest pain. Among patients receiving an Amulet device who developed an LPE, 12/99 patients (12.1%) were reported to have undergone recapturing of the device during the index procedure whereas no recaptures were reported in the LPE occurring in the Watchman group.

**Table 3:** Most Common Presentations of Late-Presenting Pericardial Effusion

	Amulet (n = 99)	Watchman (n = 2)
Chest pain	13 (13.1%)	2 (100%)
Tamponade	5 (5.1%)	0 (0.0%)
Shortness of breath	4 (4.0%)	0 (0.0%)
Routine echocardiogram	2 (2.0%)	0 (0.0%)
Routine CT	1 (1.0%)	0 (0.0%)
Dizziness	1 (1.0%)	0 (0.0%)
Not specified	73 (73.8%)	0 (0.0%)

CT = computed tomography.

## Discussion

In this study we investigated reports of PE data following LAAO with either the Amulet or Watchman devices that were submitted to the MAUDE database. We identified that a greater number of PEs were reported to the MAUDE database for those who underwent LAAO with an Amulet device than those who underwent LAAO with a Watchman device. Most patients in the Amulet group who developed LPE required intervention.

Due to the inability to obtain the total number of devices implanted, a direct comparison of Amulet to Watchman procedures and subsequent outcomes could not be performed. It is important to recognize that the most commonly implanted LAAO device remains the Watchman device. In fact, before the Amulet device was approved for the use by the FDA in 2021, the only approved device for LAAO was the Watchman device.^8^ Even after Amulet entered the market, Watchman holds >90% of the market share as of 2023.^3^ The higher amount of PEs reported with Amulet in the context of significantly lower number of total Amulet implants compared to Watchman is important to consider.

### Mechanisms of postprocedural pericardial effusion and anatomical considerations

Proposed mechanisms of postprocedural PE after LAAO device implantation involve the anatomic proximity of the left atrial appendage (LAA) to adjacent cardiac vessels and less commonly the mitral valve.^9^ Key anatomic considerations include the PA which is situated anterosuperiorly to the LAA; the left superior pulmonary vein that is posterior to the LAA; and the triangle of Brocq and Mouchet, containing the left main, left anterior descending, and left circumflex arteries.^9^ Notably the left circumflex that sits in the atrioventricular groove is directly under the LAA. The proximity of the LAA and PA can change with dilation of both structures; dilation of the LAA is common in patients with nonvalvular AF undergoing LAAO.^1^ The mitral valve lies inferiorly to the LAA. According to a systematic review, the most common contiguous vessel injury after LAAO is PA injury.^9^ The same study found that most cases of PA injury were caused by Abbott devices (including both the cardiac plug and Amulet). As the design of the Amplatzer cardiac plug is significantly different from the Amulet device, the extrapolation of outcomes with the cardiac plug may be inaccurate to apply in contemporary practice. PA perforation was cited with a high mortality rate of 40% in the same paper. The pathophysiology of PA injury with LAAO devices most likely involves a pre-existing anatomic proximity between the PA and LAA combined with oversizing of the LAAO device. The highest risk type of LAA contact with the PA is contact with the proximal LAA due to increased vulnerability of this area to injury with the LAAO anchoring hooks/stabilizing tines. An oversized device is thought to exert significant radial forces within the LAA walls and the contiguous PA that may lead to perforation via the device anchors.

**Figure 1:**
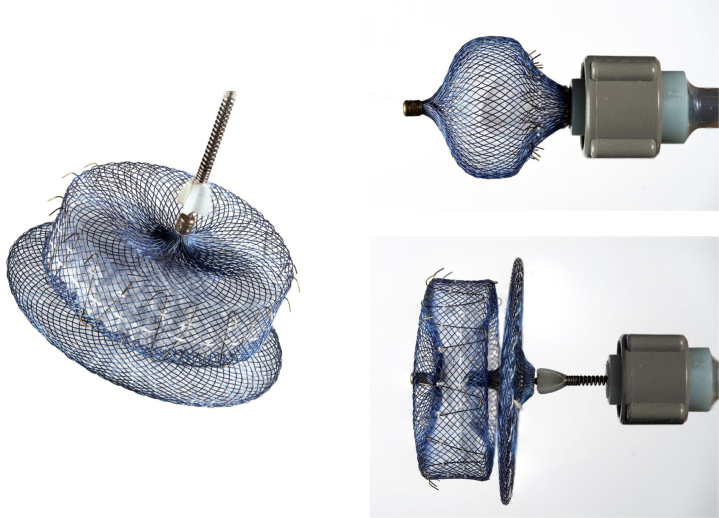
**Amulet device** The dual-sealing mechanism and accompanying stabilizing tines are shown. Image courtesy of Karl Schwarz, M.D.

**Figure 2:**
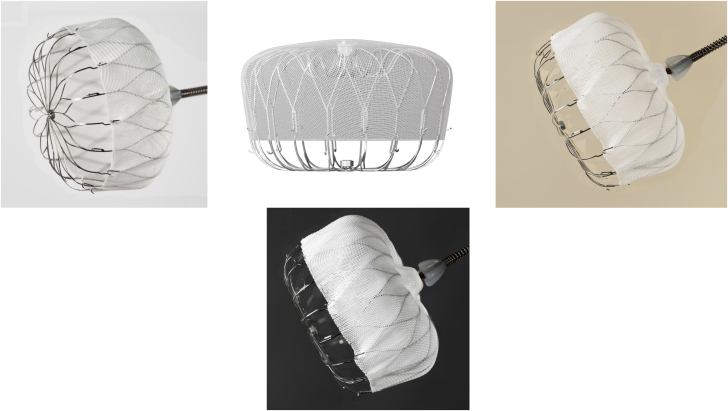
**Watchman FLX device** The single lobe sealing mechanism and accompanying device anchors are shown. Image courtesy of Karl Schwarz, MD.

**Figure 3:**
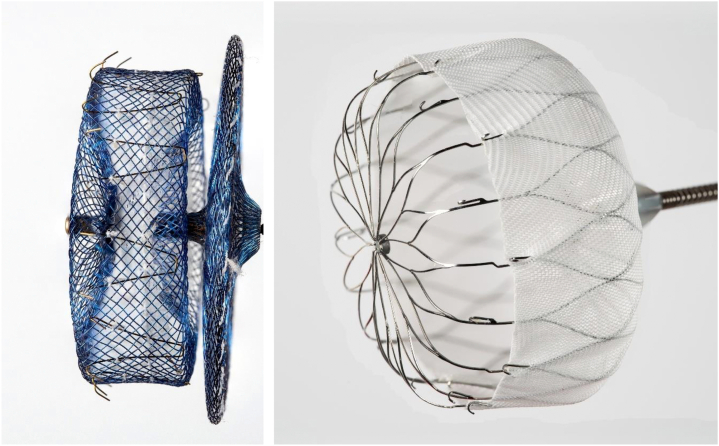
**Magnified Side-By-Side View of Amulet and Watchman FLX Device Anchors** Image courtesy of Karl Schwarz, MD.

The force of the device anchors may vary with different LAAO device design. The design differences of the Amulet device vs the Watchman primarily lie in the sealing mechanism. The Amulet device employs a dual-sealing mechanism with a lobe and a disc connected by a central waist when deployed (Figures 1A to 1C). The Watchman device utilizes a single lobe occlusion mechanism via a closed basket shape; the current iteration of the Watchman, the Watchman FLX, is shown in Figures 2A to 2D. Both device designs have stabilizing tines circumferentially around the device to secure the device into the LAA. The 2-lobed Amulet system is reported to have increased stiffness than the Watchman single-lobed system.^10^ The length of the stabilizing tines are longer in the Amulet device than the Watchman, and this may play a role in device interaction with vascular structures as discussed previously (Figure 3). Multiple studies report increased incidence of PE in the Abbott dual-seal LAAO devices over Watchman, and the proposed explanation involves the aforementioned design differences.^10^

**Figure 4:**
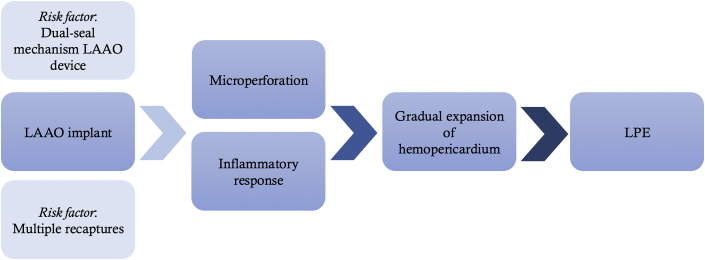
**Proposed Mechanism of Late-Presenting Pericardial Effusion** Microperforation and inflammatory response are implicated in the development of LPE. LPE = late-presenting pericardial effusion; LAAO = left atrial appendage occlusion.

The mechanism of LPE occurring weeks to months after LAAO implant is unclear. Some reports hypothesize the creation of a microperforation during implantation that results in subclinical hemopericardium.^11^ Other possible etiologies may include inflammatory effusion from increased radial forces in a dilated left atrium. In the setting of previously identified risk factors, microperforation and inflammation after device deployment is a probable mechanism of LPE (Figure 4).

### Procedural complications associated with LPE

Early insights from the EMERGE-LAA trial, which reported events up to 45 days postimplant, found an APE incidence of 1.3%, with APE defined as occurring in hospital.^12^ Pericardial effusions that were found at 45 days postimplant had an incidence of 1.9% in the same study. Of these, 0.5% required percutaneous or surgical intervention.^12^ The definition of LPE as occurring just 45 days postimplant in that study is a significant limitation as there have been multiple reports of LPE occurring several months after implant, and therefore the interpretation of this incidence is limited and not generalizable to all LPE. In addition to LPE incidence, the association of LPE with other procedural complications is not well-reported. A retrospective National Cardiovascular Data Registry (NCDR) study involving patients who received the Watchman LAAO device reported that in patients who had an in-hospital PE, stroke occurred in 0.61% and systemic embolism in 0.45% at 45-day follow-up.^13^ Data on LAAO procedural complications associated with LPE are lacking, and this is an area for increased study.

Notably, injury to the PA was reported in 12 patients who received an Amulet LAAO device in this study. An analysis from the NCDR LAAO registry of patients who underwent Watchman LAAO showed occurrence of APE in 1.35% of patients and this was associated with adverse events and increased mortality.^13^ The incidence of LPE and outcomes were not reported in that study.^13^ The distinction of in-hospital and late presentation has clinical implications on postprocedure decision-making including optimal duration of observation, need for and timing of postprocedure imaging and follow-up. Computed tomography (CT) imaging is utilized for LAAO planning and follow-up.^14^ Technical feasibility of Amulet LAAO often includes CT. Analysis of structures surrounding the LAA including proximity of the device to the PA may identify those at risk of PE after Amulet LAAO.^15^ Pracon et al^6^ identified high-risk anatomic features for PA perforation in postprocedure CT. The “cuddling lobe orientation”, proximity of the PA to the middle and distal device lobes, was considered a high-risk feature for PA injury with 2 patients suffering LPE, 1 of whom required surgery confirming PA injury.^6^ Kulawiak-Galaska et al^15^ examined the anatomical relationship and distance between the LAA and PA by CT in both the Amplatzer Amulet LAAO and the Amplatzer cardiac plug. They reported the time from device implantation to PA perforation varied from 3 hours to 6 months.^15^

**Figure 5:**
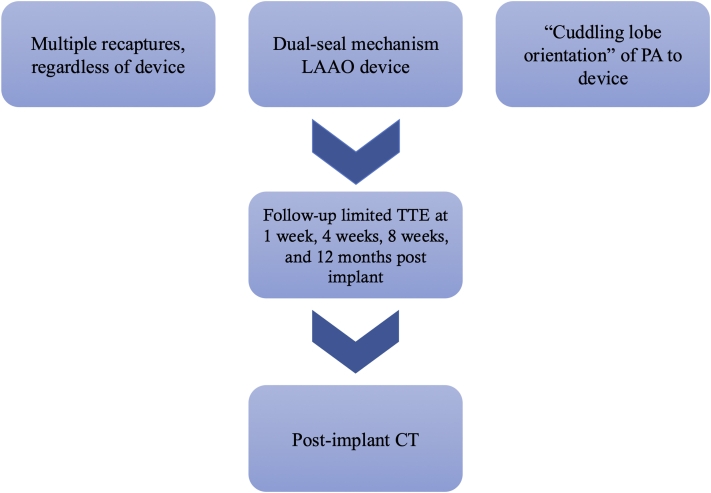
**Proposed algorithm for Late-Presenting Pericardial Effusion surveillance** This algorithm highlights postprocedural echocardiogram follow-up. PA = pulmonary artery; CT = computed tomography; TTE = transthoracic echocardiography; other abbreviation as in Figure 4.

The similarity in mortality rate between LPE and APE in those who received the Amulet LAAO implant may involve the type of preprocedural antithrombotic regimen.^10^ Galea et al.^10^ reported characteristics of PEs that occurred within 1 year after LAAO. All patients who developed LPE were discharged on dual antiplatelet therapy, whereas the antithrombotic strategy was variable preimplant—warfarin, single antiplatelet therapy, and direct oral anticoagulants were interchangeably used. Although the presence of any oral anticoagulant was associated with PE in that study, warfarin constituted a large portion of the population at 43 percent in that study. Therefore, the use of warfarin before the procedure may confer a theoretical mortality risk for APE. Multiple recaptures have been reported as an independent risk factor for PE in Abbott dual-seal LAAO devices in the same study.^10^ Multiple recaptures in a dual-seal LAAO device which has longer stabilizing wires may explain the similar observed mortality rate in both groups regardless of time course, as the mechanism for injury remains the same. Notably this study included patients who received the Abbott cardiac plug implant, which is no longer used in contemporary practice, in addition to patients who received the Amulet implant. Accordingly in our study, among patients with Amulet LAAO device implants who developed LPE, 12/99 patients (12.1%) had recaptures during the procedure. However, a potential confounding factor of increased incidence of PE with the Amulet device may be that Amulet is more often used for challenging, atypical LAA anatomy, which itself is an anatomical risk factor for procedural complications.^10^ A proposed algorithm for late-presenting pericardial effusion surveillance is shown in Figure 5.

### Study limitations

This study has limitations primarily due to the nature of the MAUDE database reporting system. The data reported are retrospective and not standardized; descriptions are of variable detail. Operator experience, medication use including anticoagulants, and comorbidities are not reported. Knowledge of the patients’ comorbidities is useful to understand the severity of patients’ baseline diseases and vulnerability. The lack of standardization of the reported data confers a limitation on the interpretation of results including possibility of confounding factors such as medication use. In the Amulet IDE trial,^16^ procedure related complications largely due to PEs were 4.5% and were more frequent in those discharged on oral anticoagulation. This study also suggested that complications decreased with operator experience. These findings were similar to what was reported in early insights from EMERGE-LAA which stratified outcomes by operator experience.^6^ In the Amulet IDE trial, PEs were reported to have resolved without sequelae with none requiring emergency surgery or resulting in death. This is in contrast to the present analysis. In addition, the MAUDE database contains only reports those that physicians elect to submit and thus the true incidence is likely under-reported.^17^ The retrospective nature of the data set confers its own limitation to these data. Due to elective reporting, bias on the part of the implanting physician to report only severe or hemodynamically significant PE may cause further underreporting of events.

Abbott Medical was contacted for the request of the total number of Amulet LAAO device implants, and the number of total implants performed was not feasible to be reported.

### Future directions

Meaningful large registry information for a low frequency complication between devices could be potentially obtained from NCDR-LAAO. Future directions for research of PE after Amplatzer Amulet LAAO could include analysis of APE and LPE incidence and outcome from the NCDR LAAO registry, prospectively investigating how anatomic features from CT imaging, such as distance from the device lobe to the PA, may influence risk for PE, and studying other LAAO procedural complications associated with LPE.

## Conclusions

In this study, the MAUDE database was reviewed to assess for PE following Amplatzer Amulet LAAO and Watchman LAAO. A greater number of postprocedural PEs were reported in Amulet patients (290) than Watchman devices (33) in the MAUDE database, and a higher amount of LPE were reported in Amulet. Periprocedural PE following LAAO can have significant morbidity and mortality (Table 1 and 2). These findings are novel and in contrast to the existing large prospective randomized controlled trials that have been performed on LAAO devices which report no morbidity and mortality associated with postprocedural PE and is shown in the Central Illustration. Further studies are needed to fully understand the incidence of LPE and that of PEs caused by PA injury following LAAO.

**Central illustration:**
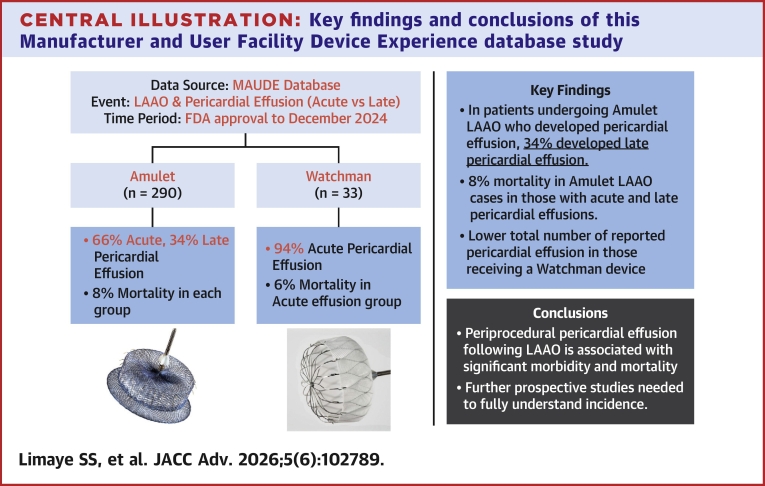
**Key findings and conclusions of this Manufacturer and User Facility Device Experience database study** Findings suggest that the morbidity and mortality of pericardial effusions following LAAO can be significant. LAAO = left atrial appendage occlusion; MAUDE = Manufacturer and User Facility Device Experience; FDA = Food and Drug Administration.

## Funding support and author disclosures

Dr Aktas has received fellowship grant support from Boston Scientific and Abbott. All other authors have reported that they have no relationships relevant to the contents of this paper to disclose.
